# Can female guppies learn to like male colours? A test of the role of associative learning in originating sexual preferences

**DOI:** 10.1098/rspb.2022.0212

**Published:** 2022-04-13

**Authors:** Magdalena Herdegen-Radwan

**Affiliations:** Department of Behavioural Ecology, Faculty of Biology, Adam Mickiewicz University in Poznan, Poznan, Poland

**Keywords:** *Poecilia reticulata*, associative learning, sexual preferences, ornaments

## Abstract

How do female sexual preferences for male ornamental traits arise? The developmental origins of female preferences are still an understudied area, with most explanations pointing to genetic mechanisms. One intriguing, little-explored, alternative focuses on the role of associative learning in driving this process. According to this hypothesis, a preference learned in an ecological context can be transferred into a sexual context, resulting in changes in mating preferences as a by-product. I tested this hypothesis by first training female guppies to associate either orange or black colour with food delivery; I then presented videos of males with computer-manipulated coloured spots and measured female preference towards them. I also allowed females from both treatments to mate with males differing in their ratio of orange-to-black spots and measured the males' reproductive success. After training, female sexual preferences significantly diverged among treatments in the expected direction. In addition, orange males sired a greater proportion of offspring with females food-conditioned on orange compared to those conditioned on black. These results show that mating preferences can arise as a by-product of associative learning, which, via translation into variation in male fitness, can become associated with indirect genetic benefits, potentially leading to further evolution.

## 1. Introduction

Female sexual preferences have long been recognized as one of the main sources (next to male–male competition) of sexual selection operating on male phenotypes [1]. However, the mechanisms that generate such preferences in the first place are still an understudied area within the field of sexual selection. It has been hypothesized [2] that natural selection would favour preferences for naturally selected traits associated with higher fitness, leading then to the elaboration of the preferred trait in a runaway process [2] or via handicap mechanism [3,4]. Alternatively, under non-adaptive scenarios, preferences could initially appear by drift [5] or evolve by a correlated response to natural selection acting on the sensory system in a non-mating context ([6,7], reviewed in [8]). What all of the above hypotheses have in common, however, is the assumption that mating preference is an evolved trait.

One viable alternative to these scenarios is associative learning: a preference for a stimulus that arises in a non-mating context, which can then be transferred (generalized) into a mating context. For example, a learned association of a particular colour with food could lead to sexual preference for male traits of that colour. The key point here is that the preference arises as a consequence of learning, so the process does not require evolved changes, but is based on plastic responses (see [9] for review on the role of learning in the evolution of new communication signals). This, it should be noted, does not preclude the action of adaptive mechanisms at further stages of preference evolution [10,11]. As reviewed by Morand-Ferron [12], associative learning is often heritable and variation in this ability could underpin genetic variation in preferences if the speed or accuracy of learning facilitate the association of environmental stimuli with male traits. This can set the stage for the runaway process, linking the preferences and preferred trait in disequilibrium, or create constant pressure on the evolution of honest ornamental traits.

Because associative learning has been reported in an extremely broad range of taxa (e.g. [12–14]), transferring learned preferences to a mating context could be a very general mechanism by which mating preferences arise. However, this mechanism has been very poorly explored. To my knowledge, the only direct test of the role of associative learning in originating female sexual preferences was reported by Amcoff & Kolm [15] in swordtail characin, a fish species in which females show feeding behaviour towards fruit-mimicking male ornaments. The authors showed that, following conditioning on differentially coloured food, females changed their respective preferences towards the colour of male ornaments.

Here, unlike in the study by Amcoff & Kolm [15], I tested the role of associative learning in originating female mating preferences for an ornament with no immediate link to feeding, as female guppies do not perform feeding behaviour in response to those ornaments and the ornaments themselves do not imitate any specific item, as is the clear case in the swordtail characin. To do so, I used the guppy (*Poecilia reticulata*), a model organism in sexual selection studies [16–19]. Males in this species possess conspicuous ornaments in the form of orange, black and iridescent spots, for which females have been shown to have sexual preferences, but no feeding reactions, in numerous populations (e.g. [16,20,21]), including the population from Tacarigua river ([22], preference for orange), from which the laboratory colony studied here was started. Guppies have also been shown to have an enhanced ability to learn colour discrimination compared to other types of discrimination [23,24]. They have been proposed as a candidate example of ecologically induced preference transferred into a mating context. Rodd *et al.* [25] showed that, in a non-mating context, guppies of both sexes are more attracted to orange objects than to objects of other colours. They suspected that this preference might have been triggered by orange fruits of the cabrehash tree (*Sloanea laurifolia*), a relatively rare plant distributed in the same areas as the guppy, as they observed high levels of competition among guppies for these protein-, sugar- and carotenoid-rich fruits fallen into streams. This observation hints to a possible link between foraging and ornamentation. Another hint comes from the result that variation in attraction to orange objects explained almost all variation in female guppy preference for orange male ornaments across populations [25]. However, the cause-and-effect relationship underlying these phenomena was unknown, and it was also not clear if the observed preferences were evolved or learned. To directly test the possible role of associative learning in shaping female preferences without the need for their evolution, here I tested if an environmentally induced preference for colour in guppy females, induced by food conditioning, would translate into sexual preferences in a mating context. In addition, I tested the fitness effects of such a transition on the ornamented males, which to my knowledge have not been previously investigated.

I trained guppy females to associate either orange or black colour with food delivery. I then created videos in which male guppies were manipulated to have spots that were artificially coloured orange, black or an uneven mixture of these colours, and used these to test the mating preferences of the experimental females. Finally, the trained females were mated to pairs of males who differed in the proportions of their orange-to-black spot areas, to test the effect of a female's training on the paternity share of her offspring. If associative learning plays a role in originating female mating preferences, I expected females conditioned on orange or black colour in a non-mating context to show preference for males with orange or black spots, respectively (or spots predominantly of the respective colours). Furthermore, if such modified preferences affect the relationship between a male's colour and his reproductive success—thus creating room for indirect selection on preferences—I expected males with mostly orange or black spots to sire more offspring with females conditioned on orange or black colour, respectively.

## 2. Methods

### (a) Study population

Experimental fish came from a laboratory population established in 2010 and were descendants of wild-caught guppies collected in 2000 from the Tacarigua river in Trinidad and reared in the laboratory of Andrea Pilastro (University of Padova, Italy). Our stock population is kept in 100 l tanks in stable conditions: water temperature of 25 ± 1°C, an alternating light/dark regime every 12 h, density of *ca* one fish per 1 l and feeding twice per day (once with live brine shrimp nauplii (*Artemia* spp.) and once with generic fish flakes).

### (b) Conditioning on colour

In each of the six blocks of the experiment, 20 virgin, sexually mature females were trained to associate one of two colours, black or orange, with food delivery. As guppies are social animals and are stressed when isolated, the females were kept in pairs, with each pair occupying one of the 10 training aquaria (15 × 30 cm). Within each pair, there was always one large (older) and one small (younger) female, which enabled visual identification by size. Each aquarium was surrounded by white walls, so as to minimize disturbance from external factors, including colours. Ten fish (in five aquaria) were conditioned on orange colour, and the other 10 on black (these are referred to as treatments from now on). The first training phase lasted 13 days and consisted of two training sessions per day, performed at least 4 h apart. During each trial, a black or orange sheet of paper, according to the treatment, was gently placed on one end of the aquarium, so that it adhered to the aquarium wall from the outside (electronic supplementary material, figure S1). Transparent sliding doors on both ends of the aquarium were then lifted by pulling a string. These doors were kept closed for the remainder of the time, so that when starting a trial, no fish was accidentally swimming in the zone nearest the end that was reserved for feeding. Immediately after the doors were lifted, one drop of pure water and one drop of water with *Artemia* larvae were released from pipettes into the aquarium: *Artemia* was released on the end with the sheet and pure water on the opposite end. This ensured that the response of the fish was to the colour of the sheet, not to the image of a pipette above the aquarium. The side the sheet was placed on was randomized for each aquarium during the experiment in order to avoid conditioning on the side of the aquarium instead of the colour. After the release of liquid, the fish were left undisturbed for 10 to 4 min. (Training started with 10 min; the time was shortened as the training phase progressed and the females took less time to approach the food and start eating. This was done to avoid displaying the sheets beyond the time the fish were feeding, which could negatively affect the effectiveness of conditioning.) After this time, the sheet was gently removed from the aquarium and the partitions lowered back in place. In order to avoid stressing the fish during the trial, a plank partition separated the aquaria from the experimenter, who inserted the tips of the pipettes through holes in the partition. In order to monitor fish behaviour and adequately adjust trial duration, fish were observed through a video camera (Microsoft LifeCam Studio Q2F-00018) placed above the aquaria. In the second phase of conditioning, which lasted for another 3 days, I presented sheets of both colours to all fish. I placed the orange and black sheets at opposite ends of the aquarium, but still provided food near the colour assigned to the conditioning treatment. I introduced this change to place the experiment in a more realistic context; in the wild, animals are confronted with a range of colours, but only some may evoke a positive reaction due to, e.g. association.

### (c) Male guppy computer animations

The videos used in the preference tests were created, animated and displayed in FishSim Animal Toolchain [26], a program dedicated to performing preference tests in fish using self-prepared animations based on manipulated photographs of real fish. The software has been previously validated in mate-choice experiments using a different poeciliid species [27,28]. I used a picture of a guppy male from the side as the basis for two sets of models, manipulated using GIMP software [29]. One set contained males in which 100% of the spots were a single colour (SC), either orange or black, with five shape variants per colour that differed in the number and shape of the spots to simulate naturally occurring variation in these traits (electronic supplementary material, figure S2A). In the second set of models, the spots were coloured with a mixture of colours (MC), i.e. in the five orange-dominant models 70–80% of the total spot area was coloured orange while the remaining spots were black, with the proportions reversed in the five black-dominant models (electronic supplementary material, figure S2B). ImageJ (http://rsbweb.nih.gov/ij/) was used to measure the spot areas. The range of total relative (to body size) spot areas used in the experiment (8–19%, mean = 10%) was within the range in the whole population (0–19%, mean = 7%), and the shift towards higher minimum areas was dictated by the nature of the experiment. The hue of the colours used for both, the sheets used during conditioning and male models, were in the range commonly recognized as orange (*ca* 15–45°; [30]), and black (close to 0°), and matched the ranges represented by the real males used in the mating trials. I recorded two videos which differed in length and in the path travelled by the fish (which can be moved around the virtual aquarium using a joystick). The first video (electronic supplementary material, movie S1) lasted 3 min; during this time, the male stayed in the centre of the aquarium and moved only slightly, swinging his fins, so as to be visible to the female in the cylinder. This video was used in the acclimation phase of the preference test (see below). The second video (electronic supplementary material, movie S2) lasted 5 min; during this time, the male moved around the whole virtual aquarium (which was limited to the field of view of the female), turning, changing direction, and going up and down. This video was used in the main part of the preference test (see below). The software makes it possible to match a recorded video with any existing model, so for each preference test a pair of orange (or orange-dominant) and black (or black-dominant) models presenting the same shape variant was displayed, with both fish swimming the same pathway.

### (d) Preference tests

Female sexual preferences were tested before and after conditioning. The test apparatus consisted of an aquarium, illuminated from above with fluorescent lamp, in which the longer walls were opaque in order to avoid visual distraction from the outside. Two identical monitors (NEC MultiSync^®^ EA245WMi) were placed directly next to the ends of the aquarium and both were connected to a computer. Each tested female was placed into a transparent cylinder positioned in the centre of the aquarium, so that she could see the test arena but could not swim away. During the 3 min acclimatization period, video 1 was displayed simultaneously on both monitors, with a black (SC blocks) or black-dominant (MC blocks) male at one end and an orange (SC blocks) or orange-dominant (MC blocks) male at the opposite end. The cylinder was then lifted and video 2 of the same models was shown on the monitors, with the female now free to swim around. At both ends of the aquarium a 5 cm-wide preference zone was marked, and the time the female spent in each zone was measured during the 5 min of the test. The whole trial was recorded using a camera placed above the aquarium in case measurements needed to be verified (e.g. due to human error). Immediately after the test, the female was returned to her home aquarium. The order in which the females were tested, the shape variant of the male model used, and the end at which orange and black models were displayed were all randomized. Each female was tested once on 2 consecutive days starting from the day after the conditioning procedure ended, i.e. twice in total. Females from the first three blocks were tested (both before and after conditioning) with SC male models, while MC models were used for testing females from blocks 4–6 (preferences after were measured in all blocks, but preferences before, for logistical reasons, were measured only in block 6).

### (e) Mating

To choose males for mating with the experimental females, 100 males from the stock population were photographed under light anaesthesia (MS-222), and the areas of their orange and black spots were measured using ImageJ. To minimize handling time and potential harm to the fish, I decided to photograph the males from one side only, based on earlier knowledge that guppy spots tend to be symmetric and that the limit I set for the differences between colours within the spot area (see point 1 below) was higher than the mean asymmetry reported in this species [31]. Ten pairs of males per block were chosen with the aim of maximizing the difference in their black–orange coloration. The rules for choosing males and pairing them were the following:

(1) on the individual fish, orange represented no more than 36% of the spot area on the black-dominant males, and no less than 64% on the orange-dominant males;
(2) within a pair, the area of each male's dominant colour (black or orange) was at least double that of the same colour in the other male.

Mating trials were performed for females from MC blocks (4–6), immediately after the last preference test following conditioning. One pair of males was put into each aquarium and left there with the two experimental females for 3 days to allow mating. Thereafter, the males were removed and photographed under light anaesthesia (using MS-222), and their tail-clips were taken for DNA analysis. At the first visual signs of pregnancy, each female was placed into an individual breeding chamber where she remained until parturition. After parturition, pictures were taken of each female under light anaesthesia for size measurements and a tail-clip was taken for molecular analysis. The offspring were euthanized by anaesthetic overdose soon after birth and stored in alcohol for DNA isolation.

### (f) Parentage assignment

DNA was extracted from tail-fin samples using the MagJet Genomic DNA Kit (Thermo Fisher Scientific) according to the manufacturer's guidelines. In order to assign F1 individuals to their parents, all individuals were screened for variation at seven previously described microsatellite loci, all of which are polymorphic in the studied population: KonD15 [32], G389 [33], TTA [34], TACA33 (H. Alexander, unpublished, based on *Xiphophorus* sequence GenBank no. AY258896), AGAT11 [35], G75 [33] and Pret77 [36]. DNA was amplified in two multiplex polymerase chain reactions using PCR Master Mix (Qiagen); one reaction amplified the first four loci while the other amplified the last three. One primer of each primer pair was fluorescently labelled to enable its identification. The 10 µl PCR mixture contained 5 µl of Master Mix, 0.2–0.4 µM of each primer and 20–100 ng of genomic DNA. The reaction conditions were as follows: a 15 min denaturation step at 95°C, followed by 36 cycles of 30 s at 94°C, 1 min at 52°C and 1 min at 72°C, then 10 min of final extension at 72°C. PCR products were mixed with a GeneScan LIZ500 size standard and electrophoresed on an ABI 3130xl Genetic Analyser. Genotyping was performed using the ABI software GeneMapper 4.0.

Parentage was assigned using COLONY 2.0 [37]. The full-likelihood method was used, and one run was performed, with the following parameters: high likelihood precision, polygamy allowed for both sexes, no sibship prior, probability of mother and father among candidates: 1 and 0.9, respectively. A male was considered the father of an individual if the associated probability of assignment of the putative offspring was above 0.7 (in 95% of assigned offspring this value was above 0.92, and in the remaining 5%, the probability of assignment for the chosen male was an order of magnitude higher than that of the other male). The number of offspring determined to be sired by a male was used as the measure of his reproductive success.

### (g) Statistical analyses

Hypotheses were tested using linear mixed models (LMMs) or generalized linear mixed models (GLMMs). For LMMs, the assumptions of normality of residual distribution and homogeneity of variances were tested with the functions shapiro.test and bartlett.test, respectively. Parameters in all models were estimated using the maximum-likelihood method. *p*-Values for general linear models were based on t-tests using Satterthwaite's method and for the GLMM based on type III Wald test. For all random effects, only random intercept was allowed. All analyses were performed in R [36], using packages lme4 [37], lmerTest [38], car [38] and mice [39]; plots were produced using the ggplot2 package [40].

In order to confirm effective randomness of assignment of females to treatments, I built two LMMs testing their preferences before conditioning with respect to treatment to which they were assigned. The response variable, preference before treatment, was calculated as the proportion of time spent by a female in the zone near the orange (SC blocks) or orange-dominant (MC block) male (i.e. this time was divided by the total time she spent in the zones of both males). In the SC model, this response variable was logit-transformed to conform to model assumptions [39]. The fixed effects were the colour on which the female will be conditioned (treatment), female size (categorical: large or small), block, trial (there were two repeats per test set-up) and aquarium side (of the orange and black model). Male model shape variant, aquarium (nested within block) and female identity were random effects. The model for SC males was based on the data from blocks 1–3, while the one for MC males used data from block 6 (see above). Additionally, to test if the time spent near orange versus black male significantly differed before treatment in the population as a whole, an intercept-only linear model was performed for all experimental females together, with the response variable calculated as the proportion of time spent within orange male zone, from which 0.5 was subtracted. Subtracting 0.5 from each measure made the expected intercept to be zero (because under null hypothesis the expected proportion of time spent near orange males should be 0.5).

Two LMMs were built to test for the effect of treatment on female preferences. The first was fitted to the data from SC blocks (1–3); the second, from the MC blocks (4–6). The response variable, measuring preference, was calculated in an analogous way to preference before conditioning, as the proportion of time spent by a female in the zone near the orange (SC blocks) or orange-dominant (MC blocks) male (i.e. this time was divided by the total time she spent in the zones of both males), and in the model for SC trials, this variable was logit-transformed to conform to model assumptions. In both models, the fixed effects were treatment, female size (categorical: large or small), block, trial (there were two repeats per test set-up) and aquarium side (of the orange and black model). Additionally, in the SC model, preference before conditioning was added as a covariate, to control for any possible effect of individual pre-experimental preferences. In both models, male model shape variant and female identity were random effects. Aquarium (nested within block) was also added as a random effect, in order to control for the non-independence of the females within pairs (e.g. due to social learning).

As due to technical problems, the preference before conditioning was only measured in one of the three MC blocks (see electronic supplementary material, methods), this variable was not added in the main MC model. However, in order to take advantage of the data available, an additional model was built, identical to the main one with the exception of adding preference before conditioning as a covariate. The multiple imputation method was used to account for the missing values, implemented in the mice package [40], with 10 imputations (*m* = 10) and 20 iterations (maxit = 20), which resulted in sufficient convergence.

To test the effect of treatment on male reproductive success, a GLMM was used with a binomial distribution of residuals, as the response variable was the number of successes and failures in siring offspring by orange-dominated males. The matching numbers of offspring sired by orange-dominated (successes) and black-dominated (failures) males were linked with the cbind function [41]. Treatment, female size (continuous, scaled) and block were modelled as fixed effects. In order to control for the non-independence of the fish within female and male pairs (e.g. due to mate-choice copying), aquarium was added as a random effect nested within block.

## 3. Results

### (a) Female preferences

**Figure 1.**
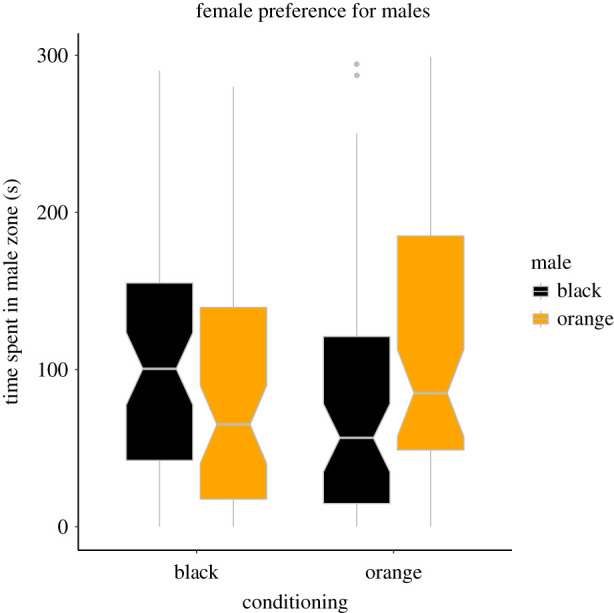
Time spent by females from orange and black treatments in the preference zone of SC male models, orange- or black-spotted. The line of the box plot shows the median; the notch represents the 95% confidence interval. (Online version in colour.)

Before conditioning, females assigned to either black or orange treatments did not differ in the time spent in the zones near differently coloured males (SC: *t* = 0.80, *p* = 0.431, *N* = 120; MC: *t* = 1.35, *p* = 0.185, *N* = 40), and no general pre-existing preference for either of the colours was detected (SC: *t* = 1.17, *p* = 0.242, *N* = 120; MC: *t* = 0.08, *p* = 0.935, *N* = 40). After conditioning, females from the orange treatment spent greater proportion of time near orange male models than black-conditioned females when tested with fully coloured males (SC blocks; *t* = 2.85, *p* = 0.008, *N* = 120; figure 1, electronic supplementary material, figure S3, full model in table 1). Given the significant effect, a post hoc test was performed to find out if the time spent near orange versus black male significantly differed within-treatments. For this purpose, an intercept-only linear model was built for each treatment separately, with the response variable calculated as the proportion of time spent within orange male zone, from which 0.5 was subtracted. These models showed that while females form the orange treatment spent significantly more time near orange males than near the black ones (*p* = 0.047), the discrimination rate of black-conditioned females did not reach significance (*p* = 0.133).

**Table 1.** LMM testing female preferences for SC simulated males. The response variable was the proportion of time spent by the female in the orange male's preference zone. The significant term is in italics.

	term	estimate	s.e.	d.f.	*t*	*p*-value
fixed effects	intercept	−0.22	0.94	100.2	−0.23	0.816
	female treatment (orange)	1.09	0.38	29.9	2.85	*0.008*
	female size (small)	0.66	0.37	87.0	1.76	0.081
	block	−0.08	0.15	29.7	−0.52	0.609
	trial	−0.32	0.37	86.0	−0.84	0.401
	male side	0.24	0.38	101.7	0.62	0.538
	preference before	0.04	0.60	119.1	0.08	0.939
random effects		variance		s.d.		
	female ID: (intercept)	0.00000		0.00004		
	aquarium: block (intercept)	0.03205		0.17900		
	male model: (intercept)	0.11970		0.34600		
residual		4.21300		2.05300		

**Figure 2.**
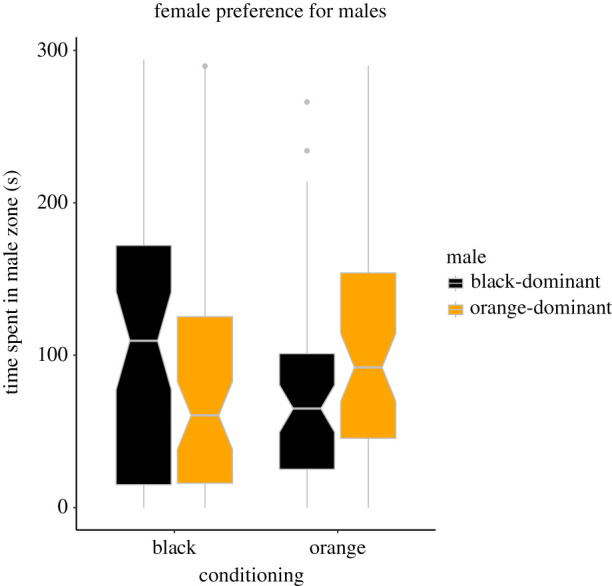
Time spent by females from orange and black treatments in the preference zone of MC male models, orange-dominant or black-dominant. The line of the box plot shows the median; the notch represents the 95% confidence interval. (Online version in colour.)

When tested with mixed-coloured males after conditioning (MC blocks), females from the two treatments tended to differ in the amount of time they spend near differently coloured males, and the effect was in the same direction as in the SC blocks, albeit it was marginally non-significant (*t* = 1.86, *p* = 0.065; *N* = 117; figure 2, electronic supplementary material, figure S4 and full model in table 2). This lack of significance may stem at least partly from decreased power of this model compared to the SC one, as the ‘preference before conditioning’ was not included due to missing data. Indeed, including this variable in an otherwise equivalent multiple imputation model resulted in a significant effect of the female treatment (*p* = 0.016; electronic supplementary material, table S1).

**Table 2.** LMM testing female preferences for MC simulated males. The response variable was the proportion of time spent by the female in the orange-dominant male's preference zone. The significant term is in italics.

	term	estimate	s.e.	d.f.	*T*	*p*-value
fixed effects	intercept	0.41	0.12	109.7	3.55	*>0.001*
	female treatment (orange)	0.11	0.61	113.4	1.86	0.065
	female size (small)	−0.01	0.61	113.3	−0.10	0.929
	block	0.01	0.03	113.0	0.589	0.669
	trial	−0.01	0.04	112.3	0.85	0.783
	male side	0.00	0.06	114.1	−0.28	0.983
random effects		variance			s.d.	
	female ID: (intercept)	0.00000			0.00002	
	aquarium: block (intercept)	0.00000			0.00000	
	male model: (intercept)	0.00123			0.03510	
residual		0.10980			0.33140	

### (b) Male reproductive success

**Figure 3.**
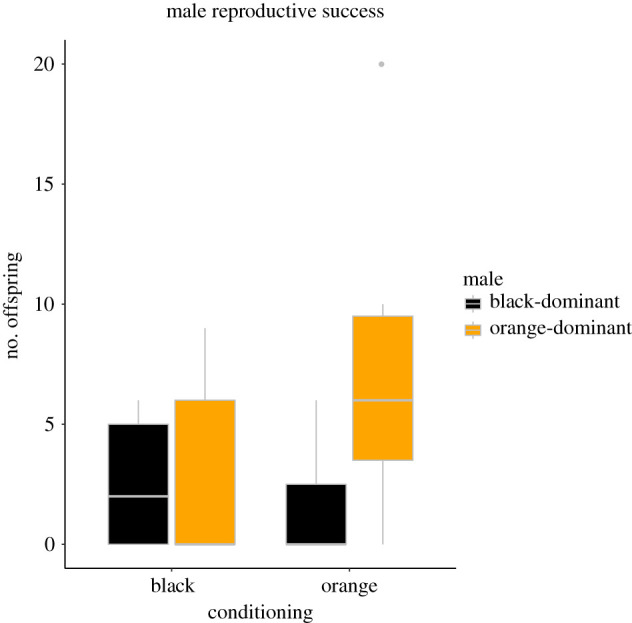
Reproductive success of orange- and black-dominant males mated with pairs of females from orange and black treatments. (Online version in colour.)

A total of 28 females (form 19 aquaria, i.e. female pairs) gave birth to 262 offspring, of which 205 (78%) were successfully assigned as the progeny of 22 males (153 and 52 assigned to orange- and black-dominant males, respectively). The paternity share of orange- and black-dominant males differed between treatments, with the proportion of offspring from orange males higher in the orange treatment (*z* = −2.77, *p* = 0.006, *N* = 28; figure 3 and table 3).

**Table 3.** GLMM testing the reproductive success of experimental males with experimental females, with female size as covariate. The response variable was the proportion of offspring in a brood that were sired by the orange-dominant male. The significant term is in italics.

	term	estimate	s.e.	*Z*	*p*-value
fixed effects	intercept	12.79	11.74	1.09	0.276
	female treatment (orange)	−18.87	6.82	−2.77	*0.006*
	female size	−0.77	0.93	−0.82	0.412
	block	−0.77	1.90	−0.40	0.687
random effects		variance		s.d.	
	aquarium: block (intercept)	212.6		14.58	

In a post hoc analysis similar to that performed for SC preference data, the intercept-only models were built, separately for each treatment. In such a binomial model, the intercept estimates the log odds ratio, and thus the associated significance level indicates whether the proportion of offspring sired by orange-dominant and black-dominant males is significantly different from 0.5. In accordance with the results from preference trials, also reproductive success differed significantly in the orange treatment, in advantage of orange-dominated males (*p* < 0.001), while the difference did not reach significance in the black treatment (*p* = 0.159).

## 4. Discussion

Associative learning offers a possible mechanism by which female sexual preferences could arise, by means of plastic—as opposed to evolved—changes in the sensory system. Learned preferences for some environmental features that arise in an ecological context can be translated into a mating context, resulting in a sensory bias towards male traits with characteristics similar to those features. Here, using an experimental approach and a simple conditioning procedure, I demonstrated that conditioning on different colours in a foraging context resulted in the divergence of preferences towards certain male ornamental traits among colour treatments, which translated into greater reproductive success for the preferred males in a competitive situation in the orange treatment.

This result is in accordance with earlier studies on swordtail characin [15,42] showing that feeding with red food resulted in an increased preference for males with artificially coloured fruit-like red flags, and for simulated red flag-ornaments. The set-up of the present experiment and the species used allowed me to detach the characteristics of the ornament from the immediate foraging context, making the interpretation of my results more general. Unlike the fruit-like flag ornament of the swordtail characin males, towards which conspecific females show feeding behaviour (biting), the coloured spots of male guppies provoke no such reaction in conspecific females. Thus, the present results reveal, for the first time, that the link between the ecologically and sexually preferred traits does not have to be immediate for associative learning to work as a trigger of sexual preferences. Also, the conditioning in this experiment was not performed on food directly, but on an associated item (the coloured sheet). My results thus suggest that the range of environmental features that may stimulate, via association, the rise of sexual preferences may be broad and go beyond those intrinsic to the preferred factor (mainly food) itself. This observation opens new perspectives in the search for possible stimuli that trigger mating preferences.

Apart from associative learning, there are other learning mechanisms that may drive sexual preferences: sexual imprinting (e.g. [43,44]) of sub-adult females on the phenotypes of adult males form the parental population and mate-choice copying [45,46] of preferences of other females. These two mechanisms involve social learning that is thought to have evolved in the mate-choice context: sexual imprinting to serve species recognition, and mate-choice copying to reduce the cost of searching for and assessing male quality and to help inexperienced females choose a mate optimally (reviewed in [47]). By contrast, the associative learning studied here does not involve social context and is unrelated to mate choice, but can nevertheless, as a side effect, influence mating decisions. It is worth noting that in the present study, there was room for behavioural copying, as females were both conditioned and then mated in pairs. While my experiment was not designed to directly study the extent of choice copying, some support for mate-choice copying comes from comparing the amount of variance explained by aquarium (linking female pairs) in the models: it was considerable and much higher in the model for mating trials than in the models for preference trials, where the same females were tested individually and thus there was no space for mate-choice copying. Importantly, however, any potential enhancement of the effect of learning due to copying or a preference bias due to mate-choice copying does not change my main conclusion, as preferences that might have been copied must have been acquired by at least some of the experimental females by associative learning.

The association tended to also affect mating preferences when females were presented with mixed-colour male models (MC, *p* = 0.065), and the significant result of the model including preference before conditioning supports the role of conditioning in inducing female preferences, suggesting that the difference between the SC and MC models may be quantitative rather than qualitative. The scenario tested in the MC models corresponds to a more realistic imitation of what females encounter in wild populations, as male guppies are almost always covered with a mixture of black and orange spots, rather than with SCs. Presented with this complex task, females still tended to discriminate among simulated males based on the dominant colour. This was further supported by the result of the mating trials, where real males covered with a mixture of both colours in different proportions were used. It is important to note here, however, that even lack of effect of conditioning in the MC preference trials would not change the main conclusions stemming from the SC part of the present study, namely that food conditioning may lead to female preferences for male traits, either the existing ones, or the ones that may arise via mutation (e.g. males with patterns in only one colour in guppies in the context of the current experiment).

For the association to arise in natural environment, several conditions have to be fulfilled regarding the strength and stability over time of the association between stimulus and trait, as well as the frequency at which individuals encounter the stimulus, all of which can modulate the extent to which associative learning may occur and affect a sexual preference [48]. The candidate example of ecological stimulus triggering female sexual preference across guppy populations, i.e. the orange fruits of the cabrehash tree, proposed by Rodd *et al.* [25], seems to fulfill those conditions. The colour matches that of the ornament and is a conspicuous one, so it should be easily visible in the otherwise green-grey-brownish environment; the fruits are rich source of protein, sugar and carotenoids, which should enhance the strength of the stimulus. This is supported by the observations of Rodd and colleagues, who reported high levels of competition among guppies for these fruits, to the extent that their occurrence was ‘the only thing that ever interrupted the males' persistent mating display’ [49]. Importantly, in tropical environments, the flowering times cover major part of the year which makes the occurrence of the stimuli relatively stable over time, while the trees are occurring throughout the guppy range, being both a permanent and not too common species, which further enhances its chances for evoking positive associations. Additionally, also small crustaceans, often rich in orange carotenoids, constitute the diet of wild guppies.

Crucially, in contrast with earlier studies, my results demonstrated that the learned mating preferences had a measurable impact on male fitness: male reproductive success depended on treatment, with more orange males siring significantly more offspring with females from the orange treatment. To my knowledge, this is the first report of fitness consequences arising as a result of associative learning between non-sexual conditioning stimuli and male ornaments subject to female mating preferences. This result further supports the role of associative learning in the evolution of male ornaments, that is, via selective pressure exerted by a female's learned preferences. If the ecological conditions in which a population lives persist over generations, the associative conditioning, e.g. on food colour, will occur in each new female generation. The generalization of learned food colour preferences to male colour traits will lead to directional selection for the preferred colour of the male ornament even in the absence of any genetic component of the preference itself.

Post hoc analyses suggested that in both the preference and mating trials the differences between treatments were driven mostly by the effect of conditioning on the orange-conditioned females. One explanation for this result may be associated with higher colour sensitivity for orange and red than for black spots (see [50] for discussion on differences in colour sensitivity in fish), making conditioning on the latter colour more difficult. The asymmetry in the impact of treatment on pre-copulatory preferences may have translated onto the post-copulatory ones, which showed even more pronounced asymmetry. Additionally (or alternatively), higher efficiency of orange males in direct competition, e.g. in sperm competition [51], could have made the effect more pronounced in orange treatment, but obscure it in black treatment. Still, even if paternity share was biased towards orange males due to their intrinsic characteristics, the main result of this experiment shows that associative learning by guppy females has the potential to change this outcome. Females play an active role during mating (e.g. [52]), and their preferences acquired via associative learning translated here into at least partial control over the paternity share of competing partners, as indicated by significant treatment effect. The proximal mechanism that allowed females to influence the outcome of mating was not tested here, but guppy females have been shown to be more behaviourally responsive to males they find attractive (see [53,54]), and can additionally bias paternity towards preferred males by controlling the duration of copulation [55] and/or the number of sperm inseminated or retained [20].

Apart from its immediate effect on female preferences, associative learning also has the potential to lead to their evolution. This may occur if there is genetic variation among females in their learning abilities, or in their sensitivity to the conditioning stimuli. Both scenarios would translate into genetic variation in preferences, potentially leading to a Fisherian runaway process [2,5]: females with stronger preferences for a trait will favour males that express it at high levels and in consequence their progeny will inherit both the genes for the preferred trait and those for the female traits influencing her learned preference [44]. This leads to linkage disequilibrium between those two traits that couples their evolutionary paths. Some evidence exists for the genetic basis of learning abilities (reviewed in [56,57]), including associative learning (reviewed in [58]), and theoretical models predict that, at least under some ecological circumstances, learned preferences may indeed evolve (reviewed in [44], e.g. [59]). Among the few empirical studies that have investigated this subject, Magurran & Ramnarine [60] suggested a potential case of learned preferences that became innate in two related, sympatrically occurring *Poecilia* species. Genetic variation in the sensory system, resulting in differences in individual sensitivity to stimuli (e.g. colours) has also been reported, especially in fish (including guppy [50], e.g. [61–63]) and insects (reviewed in [64]).

In conclusion, the present study lends strong support to the hypothesis that associative learning may be a mechanism by which sexual preferences can originate. Here, these preferences translated into male reproductive success, confirming that learned preferences can lead to the evolution of male epigamic traits. The next challenge is to assess the extent to which such a mechanism operates in nature. Future studies should also investigate whether learned preferences may evolve and co-evolve with male traits, and the use of experimental evolution could be one way to address this.

## Data Availability

Raw data are available from the Dryad Digital Repository: https://doi.org/10.5061/dryad.ttdz08m19 [65]. The data are provided in the electronic supplementary material [66].
