# Correction to ‘Can female guppies learn to like male colours? A test of the role of associative learning in originating sexual preferences’

**DOI:** 10.1098/rspb.2022.0722

**Published:** 2022-04-27

**Authors:** M. Herdegen-Radwan

*Proc. R. Soc. B* **289**, 20220212. (Published online; 6 April 2022). (doi:10.1098/rspb.2022.0212)

Block effect in models presented in tables 1–3 was entered incorrectly. Corrected tables are presented below. The results and conclusions of the paper were not affected by these changes.

**Table 1.** LMM testing female preferences for SC simulated males. The response variable was the proportion of time spent by the female in the orange male's preference zone. The significant term is in italics.

	term	estimate	s.e.	d.f.	*t*	*p*-value
fixed effects	intercept	0.04	0.81	114.7	0.05	0.962
	female treatment (orange)	1.08	0.37	116.6	2.94	*0.004*
	female size (small)	0.65	0.37	116.8	1.78	0.077
	block 2	−1.20	0.45	116.9	−2.66	0.009
	block 3	−0.46	0.45	115.7	−1.03	0.303
	repeat	−0.32	0.37	115.2	−0.86	0.390
	male side	0.22	0.37	118.1	0.58	0.564
	preference before	0.14	0.58	119.4	0.24	0.811
random effects		variance		s.d.		
	female ID: (Intercept)	0.0000		0.0000		
	aquarium: block (intercept)	0.0000		0.0000		
	male model: (intercept)	0.0939		0.3066		
residual		4.0266		2.0067

**Table 2.** LMM testing female preferences for MC simulated males. The response variable was the proportion of time spent by the female in the orange-dominant male's preference zone. The significant term is in italics.

	term	estimate	s.e.	d.f.	*t*	*p*-value
fixed effects	intercept	0.29	0.12	111.6	2.53	*0.013*
	female treatment (orange)	0.11	0.60	113.4	1.91	0.058
	female size (small)	−0.01	0.60	113.3	−0.11	0.909
	block 5	0.12	0.07	113.0	1.60	0.112
	block 6	−0.11	0.14	112.5	−0.81	0.420
	repeat	0.07	0.06	112.2	1.19	0.238
	male side	0.00	0.06	114.1	0.00	0.996
random effects		variance			s.d.	
	female id: (intercept)	0.0000			0.0000	
	aquarium: block (intercept)	0.0000			0.0000	
	male model: (intercept)	0.0012			0.0347	
residual		0.1061			0.3257

**Table 3.** GLMM testing the reproductive success of experimental males with experimental females, with female size as covariate. The response variable was the proportion of offspring in a brood that were sired by the orange-dominant male. The significant term is in italics.

	term	estimate	s.e.	*z*	*p*-value
fixed effects	intercept	8.21	10.76	0.76	0.445
	female treatment (orange)	−18.66	7.39	−2.52	*0.012*
	female size	−0.75	0.94	−0.80	0.424
	block 5	0.98	10.48	0.09	0.925
	block 6	−1.18	10.82	−0.11	0.913
random effects		variance		s.d.	
	aquarium: block (intercept)	201.5		14.2	

With this, the correct values of the respective *t* and *z* statistics and *p-*value for the treatment effects are also replacing those reported in the text of the paper.

